# Bioinformatic Analysis of Key Regulatory Genes in Adult Asthma and Prediction of Potential Drug Candidates

**DOI:** 10.3390/molecules28104100

**Published:** 2023-05-15

**Authors:** Shaojun Chen, Jiahao Lv, Yiyuan Luo, Hongjiang Chen, Shuwei Ma, Lihua Zhang

**Affiliations:** 1 Department of Traditional Chinese Medicine, Zhejiang Pharmaceutical University, Ningbo 315000, China; 2 School of Pharmacy, Nanjing University of Chinese Medicine, Nanjing 210023, China; 3 Department of Food Science, Zhejiang Pharmaceutical University, Ningbo 315000, China

**Keywords:** gene expression signature, adult asthma, Mucin-5B, lovastatin

## Abstract

Asthma is a common chronic disease that is characterized by respiratory symptoms including cough, wheeze, shortness of breath, and chest tightness. The underlying mechanisms of this disease are not fully elucidated, so more research is needed to identify better therapeutic compounds and biomarkers to improve disease outcomes. In this present study, we used bioinformatics to analyze the gene expression of adult asthma in publicly available microarray datasets to identify putative therapeutic molecules for this disease. We first compared gene expression in healthy volunteers and adult asthma patients to obtain differentially expressed genes (DEGs) for further analysis. A final gene expression signature of 49 genes, including 34 upregulated and 15 downregulated genes, was obtained. Protein–protein interaction and hub analyses showed that 10 genes, including POSTN, CPA3, CCL26, SERPINB2, CLCA1, TPSAB1, TPSB2, MUC5B, BPIFA1, and CST1, may be hub genes. Then, the L1000CDS^2^ search engine was used for drug repurposing studies. The top approved drug candidate predicted to reverse the asthma gene signature was lovastatin. Clustergram results showed that lovastatin may perturb MUC5B expression. Moreover, molecular docking, molecular dynamics simulation, and computational alanine scanning results supported the notion that lovastatin may interact with MUC5B via key residues such as Thr80, Thr91, Leu93, and Gln105. In summary, by analyzing gene expression signatures, hub genes, and therapeutic perturbation, we show that lovastatin is an approved drug candidate that may have potential for treating adult asthma.

## 1. Introduction

Defined by the Global Initiative for Asthma (GINA), asthma is a heterogeneous disease, usually characterized by chronic airway inflammation [1]. The disorder is characterized by respiratory symptoms, such as wheeze, shortness of breath, chest tightness, and cough, which vary over time and in intensity, and is accompanied by variable limitations in expiratory airflow [1,2,3]. Asthma can be induced and exacerbated by various stimuli, including allergens, pollution, and cold air, as well as microbes including respiratory viruses and especially respiratory syncytial virus and influenza virus [3]. The most common asthma phenotypes include allergic asthma, non-allergic asthma, adult-onset asthma, asthma with persistent airflow limitation, and asthma associated with obesity [1].

The prevalence of asthma appears to have now reached a plateau period after decades of increasing [2]. Even so, there is still clear regional variation, with continuous increases in prevalence in many low-income and middle-income countries [2]. Overall, asthma affects around 10% of children and adolescents and 6–7% of adults, corresponding to more than 300 million people worldwide [2,4]. Asthma is thought to account for more than 1% of the total global disability-adjusted life-years lost [5]. Therefore, the underlying mechanisms of this disease are still insufficiently elucidated, and more studies are essential to develop treatments.

Asthma occurrence and development are strongly influenced by genetics [6,7]. To date, more than 100 candidate genes are associated with a risk of asthma [7]. An analysis of the gene variants associated with asthma risk from papers published in the past over the past 20 years identified VDR, IL-13, IL-17M, TLR, HLA-DRB1, β2-AR, IFN-γ, AADAM33, CD14, and CDHR3 as the top 10 genes. [7]. Many studies of microarray analysis have been carried out to identify asthma-related genetic alterations at the genomic level, and this research provides large datasets for further investigations [8,9,10,11]. 

A more detailed analysis of available genetic data might identify genes that interact with many other genes (known as hub genes) that could be candidate biomarkers, as well as molecules that potentially perturb the gene networks. This study aimed to use large genetic datasets to gain insight into pathological mechanisms and propose potential bioactive compounds for asthma. Asthma-related gene expression datasets were obtained from the public Gene Expression Omnibus (GEO) database of the National Center for Biotechnology Information (NCBI). We then identified differentially expressed genes (DEGs) and hub genes using bioinformatics tools and searched for molecules that could reverse the abnormal gene expression in adult asthma.

## 2. Results

### 2.1. Identification of DEGs

**Figure 1:**
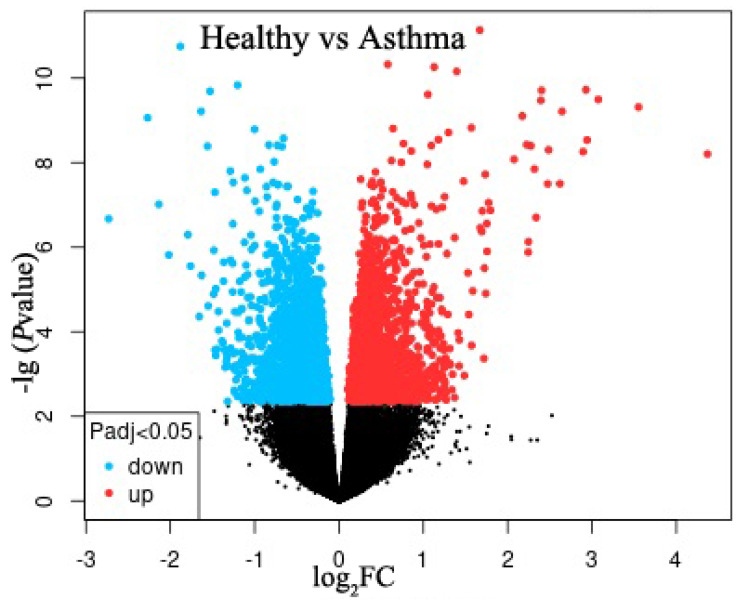
A volcano plot of the GSE179156 dataset. Red dots, upregulated differentially expression genes (DEGs); blue dots, downregulated DEGs; black dots, genes with no significant difference.

A volcano plot was used to display the gene expression profile of healthy volunteers and asthma patients from the GSE179156 dataset analyzed by the GEO2R online tool (Figure 1). According to |logFC(fold change)| ≥ 1 and adj.p-value < 0.05, 34 upregulated and 15 downregulated DEGs were identified after manual checking (Table 1).

### 2.2. Protein–Protein Interaction Analysis by GeneMANIA

**Figure 2:**
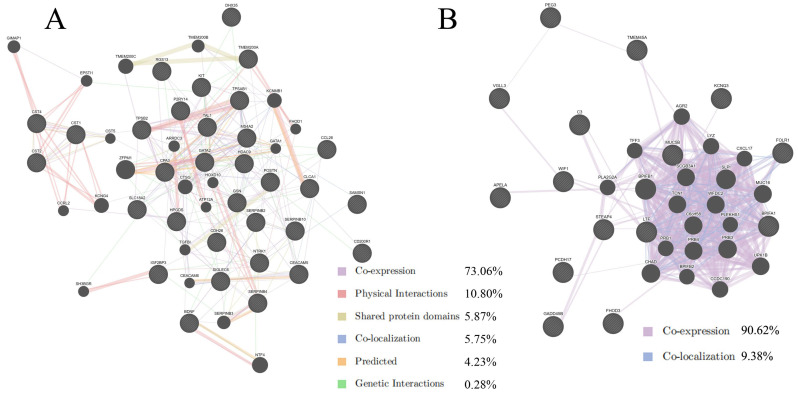
Interaction networks of (**A**) upregulated and (**B**) downregulated DEGs analyzed using GeneMANIA. Black circles, genes; colored lines, interactions between genes.

As shown in Figure 2A, upregulated DEGs displayed co-expression (73.06%), physical interactions (10.80%), sharing of protein domains (5.87%), co-localization (5.75), predicted interactions (4.23%), and genetic interactions (0.28%). As shown in Figure 2B, downregulated DEGs exhibited co-expression (90.62%) and co-localization (9.38%) interaction characteristics. Co-expression was the most common interaction in the two DEG networks (Figure 2). Co-expression represents a group of functionally-related proteins or genes highly connected by genetic and/or physical interactions, as well as genes or proteins that are members of the same molecular complex or biological pathway [12]. These results indicate that co-expressed DEGs may have the same or similar functions. GeneMANIA results also suggest that the function of upregulated DEGs mainly relates to peptidase activity regulation, and the function of downregulated DEGs mainly relates to the immune response (Supplementary Table S1). 

### 2.3. GO Enrichment and KEGG Enrichment Analyses 

**Figure 3:**
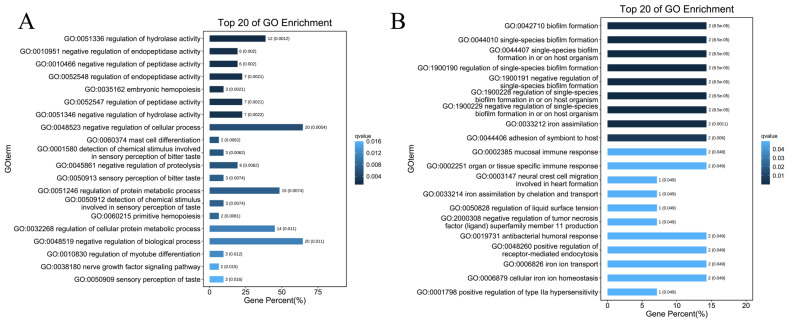
Gene ontology biological process analysis of (**A**) upregulated and (**B**) downregulated genes.

**Figure 4:**
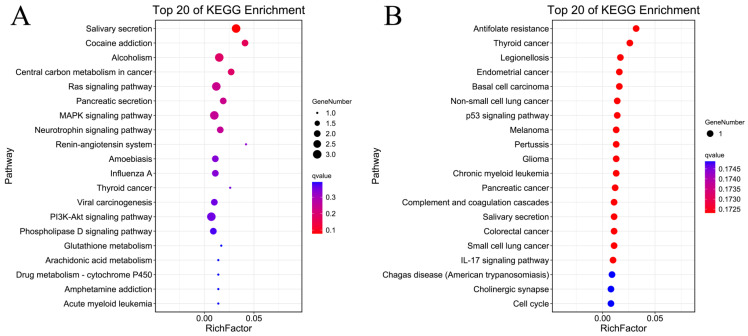
KEGG pathway analysis of (**A**) upregulated and (**B**) downregulated genes.

To better understand the function of DEGs, functional enrichment was analyzed using the OmicShare platform. Results of the GO analysis showed that upregulated DEGs were mainly enriched in regulation of hydrolase activity, negative regulation of endopeptidase activity, and negative regulation of peptidase activity (Figure 3A). The downregulated DEGs were significantly enriched in biofilm formation, single-species biofilm formation, and single-species biofilm (Figure 3B). The KEGG pathway enrichment analysis revealed that the upregulated DEGs were significantly enriched in salivary secretion, cocaine addiction, and alcoholism (Figure 4A). In addition, the downregulated DEGs were enriched in antifolate resistance, thyroid cancer, and legionellosis (Figure 4B). 

### 2.4. Identification of Hub Gene and Modules 

**Figure 5:**
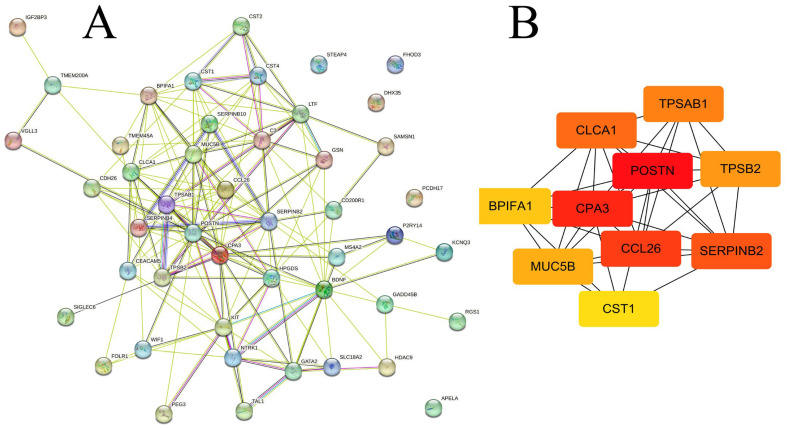
(**A**) PPIs constructed by STRING analysis. (**B**) The clustering module analyzed by the MCC algorithm.

Hubs are the principal proteins in a PPI network [13]. We constructed a PPI network for the 49 identified DEGs based on the STRING database (Figure 5A). Then, the hub genes were identified using the 12 topological analysis algorithms in the CytoHubba plugin (Supplementary Table S2). The maximal clique centrality (MCC) method in CytoHubba has a better performance than the other algorithms for identifying hubs in complex interactomes [14]. The top 10 hubs in this MCC module were POSTN, CPA3, CCL26, SERPINB2, CLCA1, TPSAB1, TPSB2, MUC5B, BPIFA1, and CST1 (Figure 5B). 

### 2.5. Drug Prediction

**Figure 6:**
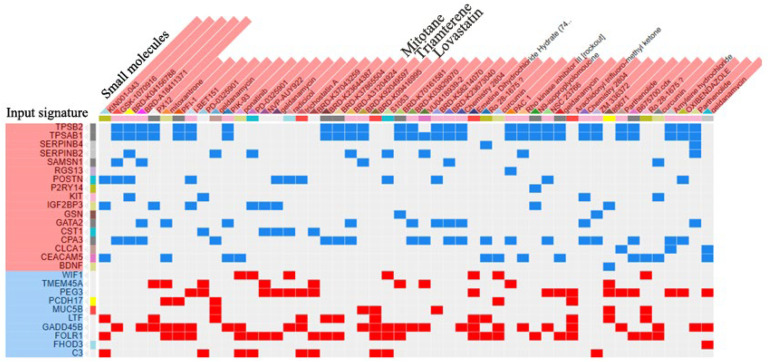
A clustergram generated from L1000 analysis showing the top-ranked molecules predicted to reverse the gene expression signature of adult asthma. Each blue cell represents an upregulated gene in adult asthma that is downregulated by the respective drug indicated at the top of the column. Each red cell represents a downregulated gene in adult asthma that is upregulated by the respective drug indicated at the top of the column.

**Figure 7:**
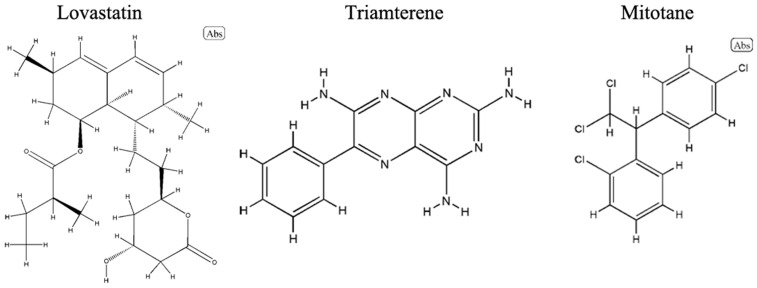
The chemical structures of lovastatin, triamterene and mitotane.

**Figure 8:**
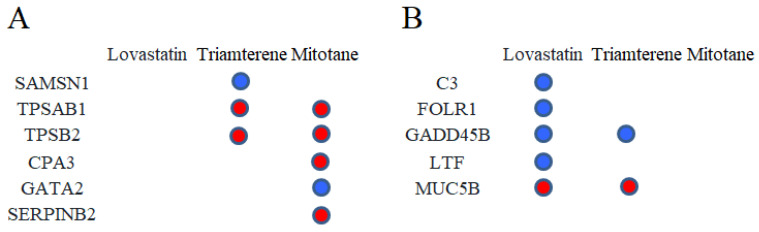
The overlap of asthma-related DEGs with the gene signatures of drug-candidate perturbation. (**A**) Upregulated. (**B**) Downregulated. Green circle indicate the overlap DEGs. Red circles represent hub genes that further intersected with MCC results.

The L1000CDS^2^ engine was used to find small molecules that could reverse the gene expression signature for asthma. A full list of these molecules is in Supplementary Table S3; the top 50 small molecules that reversed the signature were predicted according to the gene expression signature of asthma from the analysis described in Section 4.1 and are shown in the results of the clustergram analysis (Figure 6). As approved drug candidates that reverse the gene signature, lovastatin (BRD-K09416995), triamterene (BRD-K92049597), and mitotane (BRD-A31204924) had the three best scores (Figure 6). The chemical structures of the three drug candidates are shown in Figure 7. Moreover, the overlap of asthma-related DEGs with the gene signatures perturbated by lovastatin, triamterene and mitotane were shown in Figure 8. Further intersection with MCC results (Figure 5B), among the modulated targets, TPSAB1, TPSB2, CPA3, SERPINB2, and MUC5B may be the hub genes perturbed by the three drug candidates (red circles in Figure 8). 

As summarized by Drugbank, lovastatin is an HMG-CoA reductase inhibitor used to lower LDL cholesterol and reduce the risk of cardiovascular disease (Table 2). A search of the PubMed database (conducted on 18 April 2023) identified about 10 research reports about the use of lovastatin in asthma, but almost none about the use of triamterene or mitotane in asthma. Therefore, lovastatin is a compound that might perturb key genes and proteins involved in adult asthma and warrants further research as a potential treatment for this disorder. 

### 2.6. Molecular Docking Verification and Molecular Dynamics (MD) Simulation

**Figure 9:**
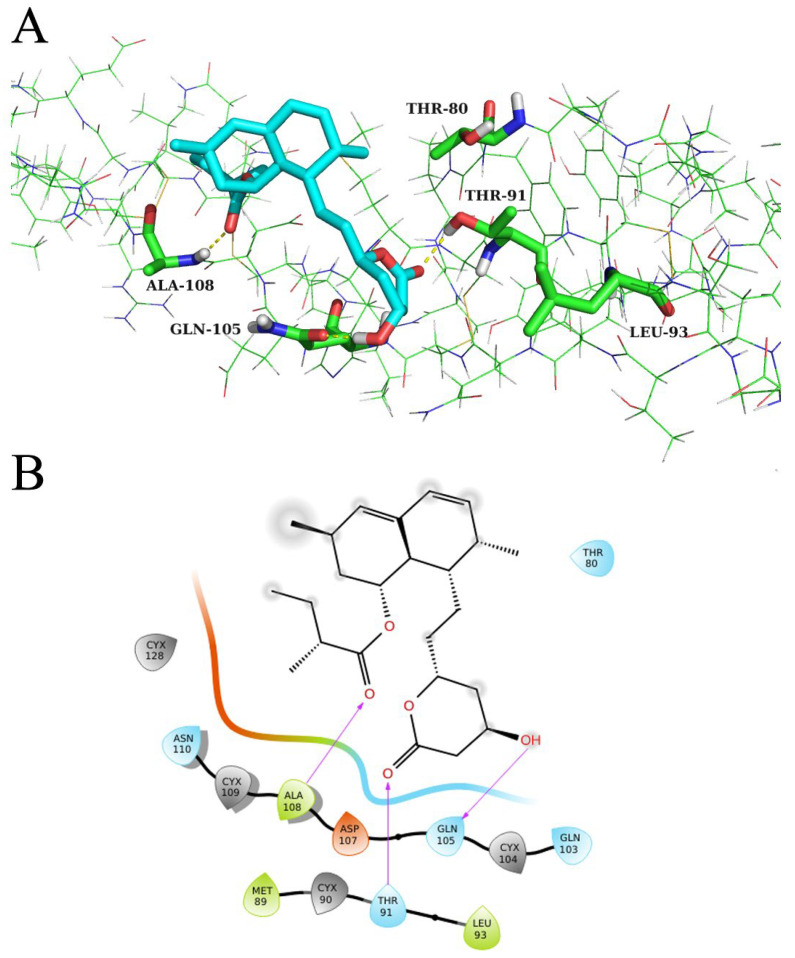
Molecular interactions between MUC5B and lovastatin shown in (**A**) 3D and (**B**) 2D.

Figure 9 shows the binding mode of lovastatin to protein MUC5B. Lovastatin can form hydrogen bonds with residues Ala108, Thr91, and Gln105 of MUC5B, and can form hydrophobic interactions with residues Leu93 and Thr80. 

**Figure 10:**
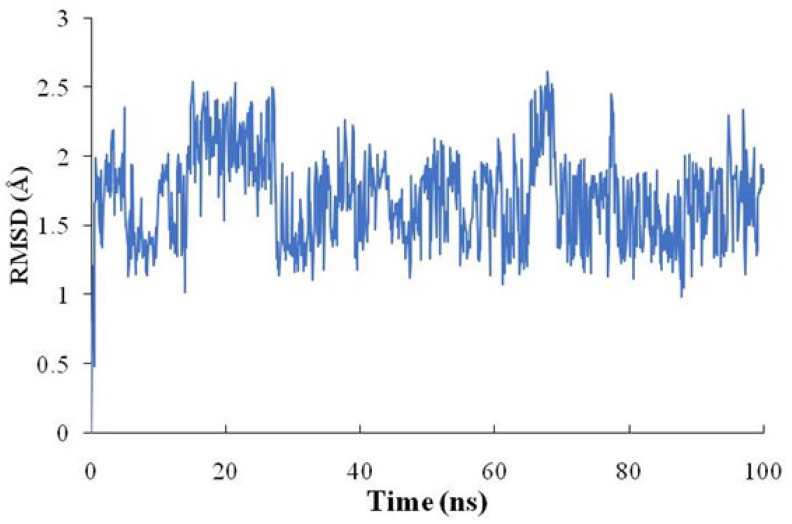
The backbone RMSD of MUC5B along the simulation time.

In addition, generally, MD results showed that the root mean square deviation (RMSD) did not fluctuate significantly during the simulation period (Figure 10). The RMSD value of the lovastatin–MUC5B complex converged to 2.0 Å, which revealed that the components of the complex steadily combine (Figure 10). 

### 2.7. Alanine Mutation of Key Residues

To further evaluate the residues that are important in the binding between lovastatin and MUC5B, a computational analysis of alanine mutation was performed. The residues Thr80, Thr91, Leu93, and Gln105 were mutated to alanine and each binding free energy was calculated. As listed in Table 3, the binding energy of wild-type MUC5B was lower than that of the mutants, indicating that the wild-type complex is more stable. The results indicated that residues Thr80, Thr91, Leu93, and Gln105 of MUC5B are important for lovastatin binding.

## 3. Discussion

Asthma is an airway disease defined by intermittent bronchospasm and is characterized by variable respiratory symptoms and variable limitations in airflow [2]. The airway epithelium is the first line of defense against pathogenic environmental factors such as allergens, pollution, viruses, and fungal and bacterial infections, and as such, it plays an important role in initiating host defense and controlling immune response [16,17]. Therefore, the airway epithelium is the central player in asthma pathogenesis [16,17]. The GSE179156 dataset used in this study represents a microarray analysis of large airway epithelium cells. Using this dataset, we identified 49 DEGs, including 34 upregulated and 15 downregulated genes, that constituted the gene expression signature for adult asthma (Table 1).

GO was developed to systematically describe and predict gene functions [18]. Regulation of hydrolase activity was the first-ranked GO biological process (GOBP) enriched for upregulated DEGs (Figure 3A). Leukotriene A4 hydrolase activation and leukotriene B4 production by eosinophils likely contributes to the presence and severity of inflammation in asthma [19]. Inhibition of soluble epoxide hydrolase reduces airway remodeling, inflammation and hyperresponsiveness, and increases epoxyeicosatrienoic acids levels in a chronic asthma model [20]. 

Biofilm formation was the first-ranked GOBP enriched for downregulated DEGs (Figure 3B). Biofilms are initially formed to facilitate the aggregation of micro-organisms, protect micro-organisms from physical and chemical stressors and environmental perturbation, and provide an efficient way to acquire and use nutrients [21,22]. Biofilm formation by dangerous infective bacteria may play an important role in the exacerbation of asthma [21]. Based on these findings, the DEGs identified in this study may participate in various biological processes important in asthma occurrence and development, such as biofilm formation and the mucosal immune response (Figure 3A,B). In addition, KEGG pathway results provide information to help understand and simulate the higher-order function of these DEGs (Figure 4A,B).

In our study, 10 biomarkers (POSTN, CPA3, CCL26, SERPINB2, CLCA1, TPSAB1, TPSB2, MUC5B, BPIFA1, and CST1) were identified as the highest-ranked hub targets; these findings are in agreement with previous research on hub genes in adult asthma (CPA3, CTSG, POSTN, CLCA1, HDC, and MUC5B) [23]. In addition, interleukins, especially IL6 and IL4, were suggested as prospective biomarkers of childhood asthma [24]. Such findings will enable us to better understand the occurrence of asthma.

Periostin was the top-ranked hub gene in the DEGs (Figure 5B, Supplementary Table S2). Periostin is a nonstructural extracellular matrix protein that binds to many extracellular matrix proteins [25]. The roles of periostin in asthma have been validated by many previous studies. Periostin expression is induced by the activity of interleukins such as IL-13, IL-5, and IL-4 in bronchial tissues, and it promotes eosinophil recruitment and adhesion in the airway subepithelial membrane of asthmatic patients [26]. Periostin activates TGF-β, PI3K/Akt, Wnt, RhoA/ROCK, NF-κB, MAPK, and JAK pathways to promote inflammation [25]. Moreover, periostin is a key driver of type-2 inflammation in asthma [27].

CPA3 was another top-ranked hub gene in the DEGs identified in this study (Figure 5B, Supplementary Table S2). It is a zinc metalloprotease that is involved in not only pathological processes but also homeostatic regulation [28]. Lung mast cells, a key type of immune cell, have higher CPA3 expression than other mast cells [28]. In a mouse asthma model, a CPA3 inhibitor reduces lung CPA activity, protects against airway hyperactivity, and reduces goblet-cell hyperplasia [29]. A previous study found that CPA3 is among the top-ranked DEGs in asthmatic subjects [30]; this finding is consistent with our results (Figure 5B, Supplementary Table S2). Even so, the role of CPA3 in asthma has not been conclusively elucidated and needs further research [31].

Gene expression signatures, such as those in the L1000CDS^2^ search engine, can be used for drug repurposing studies and to identify novel mechanisms of action for known compounds [32]. L1000CDS^2^ was used to predict compounds that reversed the gene expression signature of adult asthma (Supplementary Table S3). As shown in Figure 6 and Table 2, lovastatin (BRD-K09416995), triamterene (BRD-K92049597), and mitotane (BRD-A31204924) were the top-ranked drugs predicted to reverse the gene perturbation in adult asthma. 

Statins, also known as HMG-CoA reductase inhibitors, have immunomodulatory and anti-inflammatory properties and might be a potential therapy for asthma [33,34]. In an antigen-challenged murine asthma model, lovastatin ameliorates bronchial smooth muscle hyperresponsiveness by reducing RhoA-mediated signaling [35], and it also reduces the expression of geranylgeranyltransferase I in bronchial smooth muscle [36]. In a murine model of asthma, lovastatin reduces the infiltration of inflammatory cells into airways [35] and suppresses mucus secretion and airway inflammation by inhibiting the production of eotaxins and Th2 cytokines [37]. In samples from asthma patients, lovastatin attenuates fibroblast-to-myofibroblast transition in bronchial fibroblasts, potentially preventing bronchial-wall remodeling [38]. These findings suggest that lovastatin may have beneficial effects in asthma and are consistent with our L1000CDS^2^ results.

Moreover, the clustergram results showed there was an overlap of gene expression between the input asthma-related genes and the genes perturbated by lovastatin (Figure 6) and indicated that MUC5B was an overlapping hub gene (Figure 8B). These results indicated that MUC5B may be the target of lovastatin in asthma.

Mucins, including MUC5B, are the principal macromolecules produced by airway mucus and are crucial for maintaining the physiological functions of airways [39,40]. Altered or lower MUC5B gene expression correlates with and contributes to asthma pathogenesis [40,41]. Notably, in an ovalbumin-induced murine model of asthma, lovastatin suppresses goblet-cell hyperplasia and inhibits MUC5AC protein and gene expression [37]. Moreover, in NCI-H292 cells, lovastatin suppresses IL1beta-induced MUC5AC mRNA expression by inhibiting the p38 MAPK-dependent pathway [42]. These findings indicate that MUC5B is the likely target of lovastatin in asthma.

At present, there are no experimentally-derived crystal structures of MUC5B in the RCSB PDB database, and few structural information reports in the PubMed database. We obtained the structure of MUC5B from the AlphaFold protein structure database. The docking and MD results showed lovastatin binds with MUC5B through residues Thr80, Thr91, Leu93, and Gln105 (Figure 9 and Figure 10). Moreover, the computational analysis of alanine mutations further supports the importance of these residues (Table 3). These findings provide valuable information to further understand the MUC5B structure. Above all, lovastatin may have beneficial pharmacological effects in asthma via hub target MUC5B.

The DrugBank database contains a huge amount of openly available information about lovastatin (DrugBank ID: DB00227), such as chemical information, pharmacology, drug-product information and so on. Therefore, in addition to the use of statins as a preventive medicine for asymptomatic chronic disorders such as hypercholesterolaemia, statins might be used for acute conditions such as pain or inflammation [34]. Therefore, repurposing lovastatin for asthma is highly feasible. In summary, our L1000CDS^2^ analysis predicted that lovastatin perturbs gene networks involved in adult asthma via effects on MUC5B expression. 

## 4. Methods and Materials

### 4.1. Data Collection

The GSE179156 datasets from the GEO database for adult asthma were analyzed in this study (conducted on 7 April 2023). The GSE179156 data were collected as described previously [11] from 29 healthy controls (11 male and 18 female, aged 38 ± 11 years) and 38 asthma patients not receiving inhaled corticosteroids (25 male and 13 female, aged 31 ± 10 years). Samples of large airway epithelium cells were obtained by gentle brushing and were used for microarray analysis in the GPL570 platform [11].

### 4.2. Identification of DEGs

The R-based statistical tool GEO2R was used to identify DEGs (https://www.ncbi.nlm.nih.gov/geo/geo2r/) (conducted on 7 April 2023). GEO2R, a freely available web server, uses the limma package of R to identify DEGs in a gene expression dataset, and |logFC(fold change)| ≥ 1 and adj.p-value < 0.05 were considered statistically significant. Genes with logFC > 0 were regarded as the upregulated genes, and those with logFC < 0 were regarded as the downregulated genes. After manual checking, the upregulated and downregulated DEGs were regarded as the gene expression signature of adult asthma and used for further analysis.

### 4.3. Protein–Protein Interaction, GO Enrichment, and KEGG Enrichment Analyses

Most proteins perform a wide range of biological functions by forming a network of protein–protein interactions (PPI) [43]. The GeneMANIA website (http://genemania.org) finds functionally similar genes and predicts gene functions in an uploaded dataset using genomic and proteomic data [44]; we used this website for our analysis (conducted on 7 April 2023). Homo sapiens were chosen as the species, and the DEGs of adult asthma were uploaded for analysis. In addition, gene ontology (GO) enrichment and KEGG pathway enrichment analyses were carried out in the OmicShare platform (https://www.omicshare.com/) to provide meaningful biological functional annotation of these DEGs (conducted on 10 April 2023).

### 4.4. Hub Gene Identification

Hub genes are those with a large number of interactions in a PPI network, and they can affect the function and stability of a PPI [13]. The discovery of hub genes in various disease networks can aid the identification of molecular biomarkers [13]. In this study, a PPI network of DEGs was constructed using the STRING (https://cn.string-db.org/) database (conducted on 8 April 2023), and then the hub genes in the PPI network were analyzed by 12 topological analysis algorithms in the CytoHubba plugin [14]. The ten genes with the highest rank scores obtained in each analysis method were regarded as the hub genes.

### 4.5. Prediction of Potential Drugs 

The L1000 platform is a tool for gene expression analysis that includes 1.3 million gene-expression profiles that represent 42,080 genetic and small-molecule perturbations profiled across several cell types [45]. Therefore, the L1000 platform is a powerful tool for discovering the mechanism of action of small molecules, functionally annotating genetic variants of disease-causing genes, and finding potential ways of perturbing gene networks [45]. In this step, the upregulated and downregulated DEGs were uploaded to the L1000CDS^2^ platform (https://maayanlab.cloud/L1000CDS2/#/index), which is based on the L1000 database (conducted on 9 April 2023) [32]. Then, the top 50 chemical molecules that either mimicked or reversed the input expression profile were ranked with an overlap score and used for further investigation.

### 4.6. Molecular Docking and Molecular Dynamic Simulation

The binding mode of lovastatin and MUC5B was simulated by the Glide-Dock module of the Schrodinger Maestro 2019. Since there is no experimental crystal structure of MUC5B in the RCSB PDB database (https://www.pdbus.org/), the structure of MUC5B was obtained from AlphaFold protein structure database (https://alphafold.ebi.ac.uk/) (conducted on 15 April 2023). Then, because of the limited information on the active site in the Pubmed database, the binding cavity of MUC5B was analyzed using SiteMap tools in Schrodinger Maestro 2019. The structure file of lovastatin was downloaded from the PubChem database. 

The molecular dynamics simulation (100 ns) was performed using Desmond In Schrodinger Maestro 2019. The procedure and parameter settings were similar to those described in published research [15]. The root mean square deviation (RMSD) and binding-free energies (ΔGbind) for the complexes were calculated for further analysis.

### 4.7. Alanine Mutation of Key Residues

To confirm the active sites from the SiteMap results of Method 4.6 described above, a computational alanine scanning approach was used to identify crucial residues. Briefly, in this method, a specific residue is mutated to alanine and the difference in binding free energies before and after the mutation is computed.

Based on published research [15], the hot-spot residues of MUC5B were mutated to alanine using the Residue Mutation module in Schrodinger Maestro 2019; each mutated protein was saved. Then, after the mutated structures were prepared by the Protein Preparation module once again; MD simulation and binding free-energy calculations of the complex were performed using Desmond in Schrodinger Maestro 2019 as described above.

## 5. Conclusions

Overall, this study aimed to analyze the gene expression profile of adult asthma, find hub genes, and identify potential therapeutic molecules. We first identified 34 upregulated and 15 downregulated DEGs in the GSE179156 dataset, which is derived from a large number of healthy and asthmatic volunteers. Then, PPI and hub analyses of the DEGs showed that POSTN, CPA3, CCL26, SERPINB2, CLCA1, TPSAB1, TPSB2, MUC5B, BPIFA1, and CST1 are likely hub genes. Moreover, L1000CDS^2^ clustergram analysis of the gene expression signatures showed that lovastatin, an approved drug for lipid lowering, may perturb MUC5B expression and have the potential for treating adult asthma. Moreover, molecular docking, MD, and computational alanine scanning results supported the notion that lovastatin interacts with MUC5B via key residues, including Thr80, Thr91, Leu93, and Gln105. Obviously, the use of a bioinformatics approach to predict potential therapeutic compounds has limitations, and further wet-lab studies are needed to support these results. Nevertheless, we believe our results make an important contribution to the investigation of new genetic therapeutic targets and the selection of new drugs for treating adult asthma.

## Figures and Tables

**Table 1:** The top up- and downregulated genes in the GSE179156 dataset.

DEGs	Gene	logFC	Adj.p.Val
Upregulated	CLCA1	4.37	9.50 × 10^−6^
	CST1	3.551	2.05 × 10^−6^
	CPA3	3.075	1.54 × 10^−6^
	TPSAB1	2.941	6.70 × 10^−6^
	TPSB2	2.928	1.24 × 10^−6^
	POSTN	2.645	2.24 × 10^−6^
	SIGLEC6	2.475	3.11 × 10^−5^
	CEACAM5	2.402	1.24 × 10^−6^
	MS4A2	2.394	1.54 × 10^−6^
	SERPINB2	2.317	1.82 × 10^−5^
	CST2	2.246	2.52 × 10^−4^
	CD200R1	2.244	3.70 × 10^−4^
	RGS13	2.173	2.71 × 10^−6^
	CCL26	1.795	7.43 × 10^−5^
	SIGLEC17P	1.773	5.95 × 10^−5^
	NTRK1	1.754	1.21 × 10^−4^
	SERPINB10	1.733	2.26 × 10^−5^
	CST4	1.692	1.69 × 10^−4^
	SLC18A2	1.676	1.45 × 10^−4^
	P2RY14	1.57	4.48 × 10^−6^
	GATA2	1.396	7.60 × 10^−7^
	BDNF	1.371	2.24 × 10^−4^
	HPGDS	1.299	5.05 × 10^−6^
	SAMSN1	1.28	3.80 × 10^−4^
	DHX35	1.252	4.94 × 10^−5^
	GSN	1.227	6.83 × 10^−5^
	KIT	1.178	6.70 × 10^−6^
	CDH26	1.128	7.53 × 10^−7^
	TAL1	1.095	7.14 × 10^−6^
	TMEM200A	1.095	6.53 × 10^−5^
	IGF2BP3	1.081	2.76 × 10^−4^
	HDAC9	1.047	1.47 × 10^−5^
	SERPINB4	1.036	4.03 × 10^−4^
	PP14571	1.006	2.87 × 10^−4^
Downregulated	PEG3	−1.036	3.34 × 10^−4^
	STEAP4	−1.043	2.02 × 10^−4^
	KCNQ3	−1.093	3.83 × 10^−5^
	FOLR1	−1.116	2.67 × 10^−5^
	GADD45B	−1.121	2.56 × 10^−4^
	PCDH17	−1.254	3.03 × 10^−5^
	FHOD3	−1.259	1.22 × 10^−4^
	VGLL3	−1.286	4.63 × 10^−4^
	C3	−1.289	1.97 × 10^−5^
	LTF	−1.635	2.24 × 10^−6^
	APELA	−1.791	1.99 × 10^−4^
	TMEM45A	−1.881	4.87 × 10^−7^
	WIF1	−2.018	3.87 × 10^−4^
	MUC5B	−2.137	6.17 × 10^−5^
	BPIFA1	−2.731	1.03 × 10^−4^

**Table 2:** Information about the three drug candidates.

Perturbation	Drug Name	Drugbank ID	Summary
BRD-K09416995	Lovastatin	DB00227	Lovastatin is an HMG-CoA reductase inhibitor used to lower LDL cholesterol and reduce the risk of cardiovascular disease and associated conditions, including myocardial infarction and stroke.
BRD-K92049597	Triamterene	DB00384	Triamterene is a potassium-sparing diuretic used in the treatment of edema and the management of hypertension.
BRD-A31204924	Mitotane	DB00648	Mitotane is an adrenal cortex inhibitor used to treat adrenocortical tumors and Cushing’s syndrome.

**Table 3:** Binding free energies of MUC5B wild-type and hot-spot mutants.

Contribution	Wild-Type	T80A	T91A	L93A	G105A
ΔG_VDW_ ^a^	−27.06	−23.26	−29.62	−27.37	−25.36
ΔG_ele_ ^b^	−5.87	−3.35	−2.41	−6.85	−4.30
ΔG_GB_ ^c^	10.70	8.49	8.03	11.43	8.30
ΔG_GA_ ^d^	−39.70	−32.54	−36.15	−36.79	−31.86
ΔG_bind_ ^e^	−59.84	−49.01	−58.37	−57.90	−51.57

^a^ Contribution to the free energy of binding from the van der Waals energy [15]; ^b^ contribution to the free energy of binding from the electrostatic energy [15]; ^c^ contribution to the free energy of binding from the polar solvation energies [15]; ^d^ contribution to the free energy of binding from the non-polar solvation energies [15]; ^e^ free energy of binding.

## Data Availability

The data are freely available from the authors.

## Supplementary Materials

The following supporting information can be downloaded at: https://www.mdpi.com/article/10.3390/molecules28104100/s1. Table S1: DEG functions analyzed by GeneMANIA; Table S2: The top-ten hub genes obtained using the 12 algorithms of CytoHubba; Table S3: The top 50 small molecules that perturbed the gene signature of adult asthma identified using L1000CDS2.
