# Supplementary material for: Bioinformatic Analysis of Key Regulatory Genes in Adult Asthma and Prediction of Potential Drug Candidates
Source: Molecules. 2023 May 15;28(10):4100. doi: 10.3390/molecules28104100 (PMC10221115; doi:10.3390/molecules28104100)
Supplement: Supplementary file 1 [file molecules-28-04100-s001.zip › Supplementary Table S1.pdf]

Supplementary Table S1

DEGs	Function	FDR
Up	endopeptidase inhibitor activity	0.011172907
	endopeptidase regulator activity	0.012311225
	peptidase inhibitor activity	0.012311225
	cellular response to nerve growth factor stimulus	0.025332526
	granulocyte differentiation	0.025332526
	peptidase regulator activity	0.025332526
	response to nerve growth factor	0.072539032
	myeloid leukocyte differentiation	0.079721175
	erythrocyte homeostasis	0.085077076
	regulation of myotube differentiation	0.086863007
	neurotrophin signaling pathway	0.086863007
Down	humoral immune response	9.24E-07
	defense response to bacterium	3.42189E-05
	mucosal immune response	0.085259625
	antimicrobial humoral response	0.106437279
	organ or tissue specific immune response	0.106437279
	secretory granule lumen	0.137497276
	specific granule	0.373765116
	cell aggregation	0.373765116
	adhesion of symbiont to host	0.438013852
	regulation of toll-like receptor 4 signaling pathway	0.807796695
