# Supplementary material for: Bioinformatic Analysis of Key Regulatory Genes in Adult Asthma and Prediction of Potential Drug Candidates
Source: Molecules. 2023 May 15;28(10):4100. doi: 10.3390/molecules28104100 (PMC10221115; doi:10.3390/molecules28104100)
Supplement: Supplementary file 1 [file molecules-28-04100-s001.zip › Supplementary Table S2.pdf]

Supplementary Table S2

Rank	Algorithm											
	MCC	DMNC	MNC	Degree	EPC	BottleNeck	EcCentricity	Closeness	Radiality	Betweenness	Stress	Clustering coefficient
1	POSTN	CST2	POSTN	POSTN	POSTN	POSTN	CPA3	POSTN	POSTN	POSTN	POSTN	CST2
2	CPA3	CDH26	CPA3	CPA3	CPA3	SERPINB2	SERPINB10	CPA3	CPA3	BDNF	BDNF	TAL1
3	CCL26	TPSB2	KIT	KIT	SERPINB2	CPA3	CST1	SERPINB2	SERPINB2	CPA3	CPA3	CDH26
4	SERPINB2	MUC5B	SERPINB2	BDNF	KIT	BPIFA1	POSTN	BDNF	BDNF	SERPINB2	TMEM200A	SLC18A2
5	CLCA1	TPSAB1	C3	SERPINB2	MUC5B	BDNF	CCL26	KIT	KIT	KIT	BPIFA1	KCNQ3
6	TPSAB1	CST4	BPIFA1	C3	CCL26	TPSB2	MUC5B	BPIFA1	MUC5B	BPIFA1	SERPINB2	PEG3
7	TPSB2	CCL26	BDNF	BPIFA1	BPIFA1	VGLL3	TPSAB1	MUC5B	BPIFA1	CLCA1	CLCA1	SERPINB10
8	MUC5B	CST1	MUC5B	MUC5B	BDNF	CD200R1	LTF	CLCA1	CLCA1	LTF	KIT	TPSB2
9	BPIFA1	CLCA1	CLCA1	LTF	LTF	GATA2	C3	C3	TPSAB1	GADD45B	LTF	CST4
10	CST1	SLC18A2	CST1	CLCA1	CLCA1	TMEM200A	TPSB2	LTF	LTF	TMEM200A	GADD45B	TPSAB1
